# MCALIGN2: Faster, accurate global pairwise alignment of non-coding DNA sequences based on explicit models of indel evolution

**DOI:** 10.1186/1471-2105-7-292

**Published:** 2006-06-08

**Authors:** Jun Wang, Peter D Keightley, Toby Johnson

**Affiliations:** 1 Institute of Evolutionary Biology, School of Biological Sciences, University of Edinburgh, Edinburgh EH9 3JT, UK

## Abstract

**Background:**

Non-coding DNA sequences comprise a very large proportion of the total genomic content of mammals, most other vertebrates, many invertebrates, and most plants. Unraveling the functional significance of non-coding DNA depends on how well we are able to align non-coding DNA sequences. However, the alignment of non-coding DNA sequences is more difficult than aligning protein-coding sequences.

**Results:**

Here we present an improved pair-hidden-Markov-Model (pair HMM) based method for performing global pairwise alignment of non-coding DNA sequences. The method uses an explicit model of indel length frequency distribution which can be specified, and allows any time reversible model of nucleotide substitution. The method uses a deterministic global optimiser to find the alignment with the highest posterior probability. We test MCALIGN2 in simulations, and compare it to a previous Monte Carlo based method (MCALIGN), to the pair HMM method of Knudsen and Miyamoto, and to a heuristic method (AVID) that performed very well in a previous simulation study. We show that the pair HMM methods have excellent performance for all combinations of parameter values we have considered. MCALIGN2 is up to ten times faster than MCALIGN. MCALIGN2 is more accurate in resolving indels given an accurate explicit model than heuristic methods, but is computationally slower.

**Conclusion:**

MCALIGN2 produces better quality alignments by explicitly using biological knowledge about the indel length distribution and time reversible models of nucleotide substitution. As a result, it can outperform other available sequence alignment methods for the cases we have considered to align non-coding DNA sequences.

## Background

The advent of automated DNA sequencing methods has resulted in an enormous growth in the volume of sequence data deposited in public databases. The increasing availability of genome sequence data for many related organisms offers great opportunities to study gene function and genome evolution, but it also presents new challenges for DNA sequence analysis, especially for non-coding DNA sequences.

For much of the past two decades, research in DNA sequence analysis has focused on protein-coding sequences, which account for only a very small proportion of the total genomic content in mammals, most other vertebrates, many invertebrates, and most plants [1]. For example, protein-coding gene sequences comprise as little as 1–2% of the human and mouse genomes [2,3]. However, there is an increasing body of evidence showing that non-coding DNA sequences contain many functional sequences involved in gene regulation and potentially other unknown functions. For example, it has been estimated that ~50% of bases in intergenic and intronic sequences of *Drosophila melanogaster *are selectively constrained [4]. In rodents, it has been inferred that the total number of selectively constrained nucleotides in non-coding DNA adjacent to gene sequences is similar to that in coding DNA [5], Evidence for the presence of a large number of potentially functional non-coding sequences on human chromosome 21 has recently been obtained from a comparative genomics analysis [6]. Determining the fraction of non-coding DNA that is functional and establishing what that function is, is therefore a central problem in genome research.

Accurate inferences about the function of non-coding DNA from comparative methods depends critically on correct alignments of non-coding sequences. However, the alignment of non-coding DNA sequences is more difficult than aligning protein-coding sequences. Protein-coding sequences tend to be highly evolutionarily conserved, so insertions and deletions (indels) are uncommon and rarely cross codon boundaries. However, indel events are common in non-coding DNA, and can occur at most nucleotide sites. Numerous advances in sequence alignment methods for noncoding DNA have been made. Many recently proposed methods are based on heuristic alignment algorithms that can be very fast and accurate in cases where sequences are similar, but perform less well when sequence divergence is high [7]. Furthermore, heuristic scoring functions are not guaranteed to use the correct relationship between the relative penalties for point substitution and indel events, as they have no evolutionary interpretation. Therefore, explicit evolutionary models are desired to address this problem.

True evolutionary models of sequence evolution allow both multiple point substitutions and multiple indel events to affect any site in the sequence. The first true evolutionary model of indel evolution was introduced by Thorne, Kishino, and Felsenstein [8], the TKF91 model, and allows single-residue indel events. This method uses a maximum likelihood algorithm to estimate the evolutionary distance between two sequences, summing over all possible alignments in the likelihood calculations [8]. It was subsequently improved by allowing longer indel events with a geometric length distribution [9], by assuming that the sequence contains unbreakable fragments, and that only whole fragments are inserted and deleted. This assumption introduces hidden information in the form of fragment boundaries, and may potentially bias multiple alignment [10]. Knudsen and Miyamoto [11] presented a pairwise statistical alignment method based on an explicit evolutionary model of indel events. Indel length was assumed to be geometrically distributed, and up to two overlapping events were allowed for indels. A good approximation to such a model was then made using a pair HMM. The geometric distribution parameter, the indel rate, and the evolutionary time were estimated by maximum likelihood. A "long indel" evolutionary model has been introduced recently by Miklos et al. [12], which allows multiple-residue indels without hidden information such as fragment boundaries. They developed a finite trajectory approximation for computing the likelihood function, producing a method that has very good performance [12].

Previously, Keightley and Johnson [13] proposed a non-coding sequence alignment method called MCALIGN. This is based on a simplified evolutionary model that does not allow for any multiple hits or interaction between indel events. A key feature of their approach is that it uses additional data from "unambiguous" alignments (e.g. between sequences from closely related species) to infer the actual distribution of indel lengths, and the relative rate of indels to point substitutions. They used a Monte Carlo (MC) hill-climbing algorithm to search for the most probable alignments. This method has been successfully used for aligning real genomic sequences, such as Drosophila, rodent and hominid non-coding DNA [5,14,15]. In a simulation study, Keightley and Johnson [13] found that MCALTGN was generally superior to the other alignment methods that it was compared to.

Here, we describe an improved non-coding sequence alignment algorithm based on a generalisation of the evolutionary model used by Keightley and Johnson [13]. We show how a combination of a dynamic programming (DP) algorithm and a one dimensional deterministic optimisation – algorithm can be used to find the most probable pairwise sequence alignment. Note that when we assume the Jukes-Cantor [16] model for nucleotide substitution, the present DP method and the previous MC method are essentially using two different optimisers to attempt to maximise the same "score" function: alignment probability. However, the new optimiser is expected to be better and faster.

We have compared our method to the pair HMM method of Knudsen and Miyamoto (PairHMM_KM hereafter), which is quite similar to the present method in that it explicitly makes use of an evolutionary time parameter [11]. We have also compared our method to the heuristic alignment program AVID of Bray et al. [17] in simulations that assume a general-time-reversible (GTR) model [18] that had first been fitted to real *Drosophila *non-coding DNA sequence data. It has been shown that AVID performs very well compared to other heuristic methods [13,17], so here we only compare our method to AVID rather than other heuristic methods.

In our tests, the new DP method (MCALIGN2) is up to ten times faster than the previous MC method (MCALIGN), and is also faster than the pairHMM_KM method [11], although none can compete in speed terms with heuristic methods.

For cases of real non-coding sequence data, we also compared MCALIGN2 with AVID and CLUSTALW [19], and show that they perform differently for some specific cases.

### Implementation

We use a Bayesian statistical framework [20,21] to make inference about the pairwise alignment. The aim is to compute the posterior probabilities of different possible alignments, using the observed sequences as data and eliminating other "nuisance" parameters from the analysis. Here we focus on finding the alignment with the highest posterior probability.

Let *t *be the total divergence time between two sequences, *a *be an alignment of two sequences, and *S *be the observed data, which is two non-coding DNA sequences. In a Bayesian framework, the behaviours of all variables are modelled by probability distributions. Joint inference about *a *and *t *is accomplished simply via Bayes' theorem

P(a,t|S)=P(a,S|t)P(t)1P(S).     (1)
 MathType@MTEF@5@5@+=feaafiart1ev1aaatCvAUfKttLearuWrP9MDH5MBPbIqV92AaeXatLxBI9gBaebbnrfifHhDYfgasaacH8akY=wiFfYdH8Gipec8Eeeu0xXdbba9frFj0=OqFfea0dXdd9vqai=hGuQ8kuc9pgc9s8qqaq=dirpe0xb9q8qiLsFr0=vr0=vr0dc8meaabaqaciaacaGaaeqabaqabeGadaaakeaacqWGqbaucqGGOaakcqWGHbqycqGGSaalcqWG0baDcqGG8baFcqWGtbWucqGGPaqkcqGH9aqpcqWGqbaucqGGOaakcqWGHbqycqGGSaalcqWGtbWucqGG8baFcqWG0baDcqGGPaqkcqWGqbaucqGGOaakcqWG0baDcqGGPaqkdaWcaaqaaiabigdaXaqaaiabdcfaqjabcIcaOiabdofatjabcMcaPaaacqGGUaGlcaWLjaGaaCzcamaabmaabaGaeGymaedacaGLOaGaayzkaaaaaa@4DF5@

The probability *P*(*S*) that appears in the denominator of equation (1) may be difficult to calculate, but because in Bayesian inference the observed data *S *is held fixed, the unconditional probability *P*(*S*) is constant. We can therefore make our inference using only relative probabilities and *P*(*S*) need not be calculated. The other unconditional probability that appears in equation (1) is *P*(*t*), which is specified as a prior; our method will work for any prior.

To calculate the posterior probability of an alignment, we consider the divergence time *t *as a nuisance parameter. The posterior probability for an alignment is therefore marginal to the divergence time *t*, and is calculated using the integral

*P*(*a*|*S*) = ∫ *P*(*a*, *t*|*S*) *dt*.    (2)

We approximate this integral using Laplace's method, described in detail below.

#### Probability model of sequence evolution

**Figure 1:**
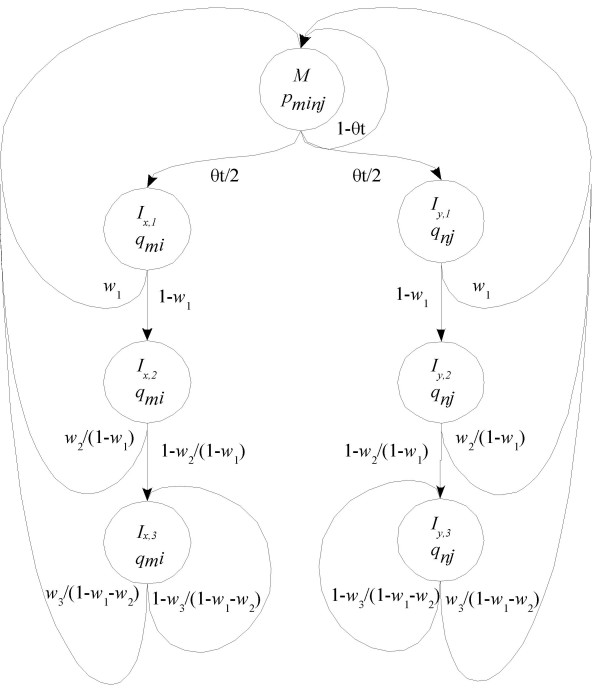
Pair HMMs assuming an affine gap model. Assume two homologous sequences *x *and *y*. Let *m*_*i *_be the *i*th nucleotide in sequence *x *and *n*_*j *_be the *j*th nucleotide in sequence *y*. *M *represents the state that *m*_*i *_is aligned to *n*_*j*_, *I*_*x *_represents the state that *m*_*i *_is aligned to a gap (in an insertion with respect to *y*), and *I*_*y *_represents the state that *n*_*j *_is in an insertion with respect to *x*. The numbers shown after *x *or *y *indicate the positions of *m*_*i *_and *n*_*j *_in the insertion with respect to the other sequence. The transition probability is shown between states.

The most difficult probability to specify in equation (1) is *P*(*a,S*|*t*), which is the joint probability of alignment *a *and sequences *S *given a divergence time *t*. This probability is specified according to a model. Here, we use the pair hidden Markov model (HMM) shown in Figure 1. For a comprehensive introduction to pair HMMs, see the books by Durbin et al. [21] and Ewens and Grant [22]. For a given time *t*, the pair HMM shown in Figure 1 generates the sequence alignment by using a series of transitions between states, accompanied by emissions. Once in a given state, the transition probabilities (shown in Figure 1) govern which state the pair HMM will move to next. Upon arrival at a new state, the pair HMM emits some observed data according to the emission probability distributions (shown in Figure 1). For example, state *M *has emission probability distribution pminj
 MathType@MTEF@5@5@+=feaafiart1ev1aaatCvAUfKttLearuWrP9MDH5MBPbIqV92AaeXatLxBI9gBaebbnrfifHhDYfgasaacH8akY=wiFfYdH8Gipec8Eeeu0xXdbba9frFj0=OqFfea0dXdd9vqai=hGuQ8kuc9pgc9s8qqaq=dirpe0xb9q8qiLsFr0=vr0=vr0dc8meaabaqaciaacaGaaeqabaqabeGadaaakeaacqWGWbaCdaWgaaWcbaGaemyBa02aaSbaaWqaaiabdMgaPbqabaWccqWGUbGBdaWgaaadbaGaemOAaOgabeaaaSqabaaaaa@3431@ for emitting an aligned base pair *m*_*i*_:*n*_*j*_, and state *I*_*x *_and *I*_*y *_have distributions qmi
 MathType@MTEF@5@5@+=feaafiart1ev1aaatCvAUfKttLearuWrP9MDH5MBPbIqV92AaeXatLxBI9gBaebbnrfifHhDYfgasaacH8akY=wiFfYdH8Gipec8Eeeu0xXdbba9frFj0=OqFfea0dXdd9vqai=hGuQ8kuc9pgc9s8qqaq=dirpe0xb9q8qiLsFr0=vr0=vr0dc8meaabaqaciaacaGaaeqabaqabeGadaaakeaacqWGXbqCdaWgaaWcbaGaemyBa02aaSbaaWqaaiabdMgaPbqabaaaleqaaaaa@3139@ and qnj
 MathType@MTEF@5@5@+=feaafiart1ev1aaatCvAUfKttLearuWrP9MDH5MBPbIqV92AaeXatLxBI9gBaebbnrfifHhDYfgasaacH8akY=wiFfYdH8Gipec8Eeeu0xXdbba9frFj0=OqFfea0dXdd9vqai=hGuQ8kuc9pgc9s8qqaq=dirpe0xb9q8qiLsFr0=vr0=vr0dc8meaabaqaciaacaGaaeqabaqabeGadaaakeaacqWGXbqCdaWgaaWcbaGaemOBa42aaSbaaWqaaiabdQgaQbqabaaaleqaaaaa@313D@ for emitting nucleotide base *m*_*i *_and *n*_*j *_against a gap, in each of the two sequences (labelled *x *and *y *respectively).

The transition probabilities for the pair HMM determine the pattern of indels in the alignment. The emission probabilities for the pair HMM determine the sequences that are observed, given the pattern of indels in the alignment. We specify the transition probabilities with an explicit model of insertion and deletion events, and the emission probabilities are specified by a model of nucleotide frequencies and of nucleotide substitutions. We consider the transition and emission probabilities in turn.

We assume that insertions and deletions occur as independent events over time with a total rate θ per interbase site relative to nucleotide substitutions. As we ignore multiple hits for indels, the probability of an indel is 1-e^(-θ*t*) ^per interbase site, which we approximated as θ*t*, an approximation that should be good for small values of *t*. An indel can correspond to a gap in sequence *x *or a gap in sequence *y*. These two events have the same probability, so the probability of a gap in either of the two sequences, *x *and *y*, is then θ*t*/2. In Figure 1, this corresponds to the transition probability from the *M *state to the *I*_*x*,1 _state, or to the *I*_*y*,1 _state. The pair HMM must move through one of these states whatever the length of the indel.

**Figure 2:**
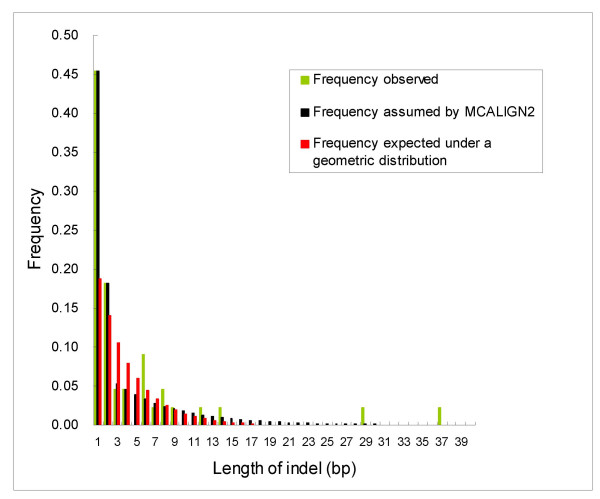
The empirical distribution of indel lengths in noncoding DNA between *D. simulans *and *D. sechellia *from Keightley and Johnson 2004 (green histogram), the indel length frequency distribution assumed by MCALIGN2 (black histogram) and the indel length frequency distribution expected under a geometric distribution based on maximum likelihood estimation given the observed data (red histogram).

The standard affine gap model corresponds to assuming that the lengths of indels follow a geometric distribution [21,23]. Empirical data on indel lengths in Drosophila non-coding DNA show an obvious departure from a geometric distribution, since 1- or 2-residue indels are more common than expected (Figure 2). Therefore, our model includes separate parameters for the probabilities of indels of length 1-bp and 2-bp, since these can be reliably estimated. Because there are less data on the length distribution for longer indels, we assumed a geometric distribution. Other more complex distributions are widely preferred for protein sequence alignments [12,24], but their large numbers of parameters cannot be reliably fitted using available data for noncoding sequences. Let *w*_*i *_be the probability of an indel of length *i*, with *w*_*i*+2_/*w*_*i*+1 _= *w*_*i*+1_/*w*_*i *_for *i *≥ 3, and ∑_*i*_*w*_*i *_= 1. We follow the approach of Keightley and Johnson [13] and estimate these parameters, along with the parameter θ that describes the total rate of indels relative to nucleotide substitutions, from additional data, as described below.

As shown in Figure 1, given that the pair HMM has arrived in state *I*_*x,i *_or *I*_*y,i *_(for any *i *≥ 1), the transition probability back to the *M *state is *w*_*i*_/(1−∑j=1i−1wj)
 MathType@MTEF@5@5@+=feaafiart1ev1aaatCvAUfKttLearuWrP9MDH5MBPbIqV92AaeXatLxBI9gBaebbnrfifHhDYfgasaacH8akY=wiFfYdH8Gipec8Eeeu0xXdbba9frFj0=OqFfea0dXdd9vqai=hGuQ8kuc9pgc9s8qqaq=dirpe0xb9q8qiLsFr0=vr0=vr0dc8meaabaqaciaacaGaaeqabaqabeGadaaakeaacqGGOaakcqaIXaqmcqGHsisldaaeWaqaaiabdEha3naaBaaaleaacqWGQbGAaeqaaaqaaiabdQgaQjabg2da9iabigdaXaqaaiabdMgaPjabgkHiTiabigdaXaqdcqGHris5aOGaeiykaKcaaa@3BC7@ and the transition probability to state *I*_*x,i*+1 _or *I*_*y,i*+1 _is 1 - *w*_*i*_/(1−∑j=1i−1wj)
 MathType@MTEF@5@5@+=feaafiart1ev1aaatCvAUfKttLearuWrP9MDH5MBPbIqV92AaeXatLxBI9gBaebbnrfifHhDYfgasaacH8akY=wiFfYdH8Gipec8Eeeu0xXdbba9frFj0=OqFfea0dXdd9vqai=hGuQ8kuc9pgc9s8qqaq=dirpe0xb9q8qiLsFr0=vr0=vr0dc8meaabaqaciaacaGaaeqabaqabeGadaaakeaacqGGOaakcqaIXaqmcqGHsisldaaeWaqaaiabdEha3naaBaaaleaacqWGQbGAaeqaaaqaaiabdQgaQjabg2da9iabigdaXaqaaiabdMgaPjabgkHiTiabigdaXaqdcqGHris5aOGaeiykaKcaaa@3BC7@. (Here a sum with no terms is understood to be zero.) This produces the desired distribution of indel lengths. We also assume that a gap in sequence *x *will not be followed directly by a gap in sequence *y*, and therefore there are no transitions from any of the states *I*_*x *_to any of the states *I*_*y*_, or vice versa. Our approach could be extended to accommodate an indel length distribution that is any mixture of geometric distributions (as used by Miklos et al. [12]) by duplicating the nodes in the pair HMM for insertions and deletions, and setting transition probabilities according to each component in the mixture. Such an extension may lead to increased accuracy, but at the expense of increased computational demands.

In order to make our pair HMMs describe a probability distribution over all possible alignments, we need to include a Begin state and an End state. We set the transition probability from the Begin state to states *M*, *I*_*x*,1 _and *I*_*y*,1 _to be the same as those from the *M *state. We allow all states to make transitions to the End state, with a low transition probability ε. If ε is small enough, we can ignore it in all of our calculations [21].

The emission probabilities, which determine the sequences given the pattern of indels, are derived from the general time-reversible (GTR) model of nucleotide substitution [18]. The Jukes-Cantor [16] model and the Kimura-2-parameter [25] model are two specific cases of the GTR model when certain parameters are fixed.

The emission probabilities qmi
 MathType@MTEF@5@5@+=feaafiart1ev1aaatCvAUfKttLearuWrP9MDH5MBPbIqV92AaeXatLxBI9gBaebbnrfifHhDYfgasaacH8akY=wiFfYdH8Gipec8Eeeu0xXdbba9frFj0=OqFfea0dXdd9vqai=hGuQ8kuc9pgc9s8qqaq=dirpe0xb9q8qiLsFr0=vr0=vr0dc8meaabaqaciaacaGaaeqabaqabeGadaaakeaacqWGXbqCdaWgaaWcbaGaemyBa02aaSbaaWqaaiabdMgaPbqabaaaleqaaaaa@3139@ and qnj
 MathType@MTEF@5@5@+=feaafiart1ev1aaatCvAUfKttLearuWrP9MDH5MBPbIqV92AaeXatLxBI9gBaebbnrfifHhDYfgasaacH8akY=wiFfYdH8Gipec8Eeeu0xXdbba9frFj0=OqFfea0dXdd9vqai=hGuQ8kuc9pgc9s8qqaq=dirpe0xb9q8qiLsFr0=vr0=vr0dc8meaabaqaciaacaGaaeqabaqabeGadaaakeaacqWGXbqCdaWgaaWcbaGaemOBa42aaSbaaWqaaiabdQgaQbqabaaaleqaaaaa@313D@ are the equilibrium frequencies of nucleotides *m*_*i *_and *n*_*j*_, which are equal for sequences *x *and *y*. The emission probabilities pminj
 MathType@MTEF@5@5@+=feaafiart1ev1aaatCvAUfKttLearuWrP9MDH5MBPbIqV92AaeXatLxBI9gBaebbnrfifHhDYfgasaacH8akY=wiFfYdH8Gipec8Eeeu0xXdbba9frFj0=OqFfea0dXdd9vqai=hGuQ8kuc9pgc9s8qqaq=dirpe0xb9q8qiLsFr0=vr0=vr0dc8meaabaqaciaacaGaaeqabaqabeGadaaakeaacqWGWbaCdaWgaaWcbaGaemyBa02aaSbaaWqaaiabdMgaPbqabaWccqWGUbGBdaWgaaadbaGaemOAaOgabeaaaSqabaaaaa@3431@ are the probabilities of starting with an unobserved common ancestor nucleotide *o*, drawn from the equilibrium distribution of nucleotide frequencies, and evolving independently down two lineages, to *m*_*i *_in time *t*_1 _along one lineage and to *n*_*j *_in time *t*_2 _along the other lineage. (Under a time reversible model, this is the same as the probability of starting with *n*_*j *_and evolving to *m*_*i *_(or vice versa) in time *t*_1_+ *t*_2_). Since the times *t*_1 _and *t*_2 _are individually nonindentifiable, we parameterise our model simply by the total divergence time *t *= *t*_1_+ *t*_2_. For a given total divergence time, the conditional probability of evolving to *m*_*i *_given starting with *n*_*j *_is pmi|nj=pminj/qnj
 MathType@MTEF@5@5@+=feaafiart1ev1aaatCvAUfKttLearuWrP9MDH5MBPbIqV92AaeXatLxBI9gBaebbnrfifHhDYfgasaacH8akY=wiFfYdH8Gipec8Eeeu0xXdbba9frFj0=OqFfea0dXdd9vqai=hGuQ8kuc9pgc9s8qqaq=dirpe0xb9q8qiLsFr0=vr0=vr0dc8meaabaqaciaacaGaaeqabaqabeGadaaakeaacqWGWbaCdaWgaaWcbaGaemyBa02aaSbaaWqaaiabdMgaPbqabaWccqGG8baFcqWGUbGBdaWgaaadbaGaemOAaOgabeaaaSqabaGccqGH9aqpcqWGWbaCdaWgaaWcbaGaemyBa02aaSbaaWqaaiabdMgaPbqabaWccqWGUbGBdaWgaaadbaGaemOAaOgabeaaaSqabaGccqGGVaWlcqWGXbqCdaWgaaWcbaGaemOBa42aaSbaaWqaaiabdQgaQbqabaaaleqaaaaa@43C7@, and the matrix of these conditional probabilities, *Q*(*t*), can be calculated from the fixed instantaneous rate matrix *A *by matrix exponentiation [26], that is,

*Q*(*t*) = *e*^*tA*^.     (3)

which can be calculated using the eigenvalues and eigenvectors of *A*. Here we estimate the rate matrix *A *from the same external data that is used to estimate the parameters for indels, as described below.

#### Alignment algorithm

Given that *P*(*a,S*|*t*) has been specified by the model, and that a prior *P*(*t*) for the divergence time has also been specified, we have developed an algorithm to infer the approximate maximum a-posteriori (MAP) alignment a^
 MathType@MTEF@5@5@+=feaafiart1ev1aaatCvAUfKttLearuWrP9MDH5MBPbIqV92AaeXatLxBI9gBaebbnrfifHhDYfgasaacH8akY=wiFfYdH8Gipec8Eeeu0xXdbba9frFj0=OqFfea0dXdd9vqai=hGuQ8kuc9pgc9s8qqaq=dirpe0xb9q8qiLsFr0=vr0=vr0dc8meaabaqaciaacaGaaeqabaqabeGadaaakeaacuWGHbqygaqcaaaa@2E07@. This is the alignment with highest posterior probability given the observed sequences, with the divergence time eliminated as a nuisance parameter. Thus, a^
 MathType@MTEF@5@5@+=feaafiart1ev1aaatCvAUfKttLearuWrP9MDH5MBPbIqV92AaeXatLxBI9gBaebbnrfifHhDYfgasaacH8akY=wiFfYdH8Gipec8Eeeu0xXdbba9frFj0=OqFfea0dXdd9vqai=hGuQ8kuc9pgc9s8qqaq=dirpe0xb9q8qiLsFr0=vr0=vr0dc8meaabaqaciaacaGaaeqabaqabeGadaaakeaacuWGHbqygaqcaaaa@2E07@ is the alignment that maximises *P*(*a*|*S*), which is given by the integral in equation (2). To approximate this integral, we assume that *P*(*a*, *t*|*S*), when treated as a function of *t *with both *a *and *S *held fixed, is approximately Gaussian. Then, using Laplace's method [27], we can write

P(a|S)≈P(a⋅t^a|S)2π|Va|12.     (4)
 MathType@MTEF@5@5@+=feaafiart1ev1aaatCvAUfKttLearuWrP9MDH5MBPbIqV92AaeXatLxBI9gBaebbnrfifHhDYfgasaacH8akY=wiFfYdH8Gipec8Eeeu0xXdbba9frFj0=OqFfea0dXdd9vqai=hGuQ8kuc9pgc9s8qqaq=dirpe0xb9q8qiLsFr0=vr0=vr0dc8meaabaqaciaacaGaaeqabaqabeGadaaakeaacqWGqbaucqGGOaakcqWGHbqycqGG8baFcqWGtbWucqGGPaqkcqGHijYUcqWGqbaucqGGOaakcqWGHbqycqGHflY1cuWG0baDgaqcamaaBaaaleaacqWGHbqyaeqaaOGaeiiFaWNaem4uamLaeiykaKYaaOaaaeaacqaIYaGmcqaHapaCaSqabaGccqGG8baFcqWGwbGvdaWgaaWcbaGaemyyaegabeaakiabcYha8naaCaaaleqabaWaaSaaaeaacqaIXaqmaeaacqaIYaGmaaaaaOGaeiOla4IaaCzcaiaaxMaadaqadaqaaiabisda0aGaayjkaiaawMcaaaaa@50AD@

Here t^a
 MathType@MTEF@5@5@+=feaafiart1ev1aaatCvAUfKttLearuWrP9MDH5MBPbIqV92AaeXatLxBI9gBaebbnrfifHhDYfgasaacH8akY=wiFfYdH8Gipec8Eeeu0xXdbba9frFj0=OqFfea0dXdd9vqai=hGuQ8kuc9pgc9s8qqaq=dirpe0xb9q8qiLsFr0=vr0=vr0dc8meaabaqaciaacaGaaeqabaqabeGadaaakeaacuWG0baDgaqcamaaBaaaleaacqWGHbqyaeqaaaaa@2FA4@ is the mode, or value of *t *that maximises *P*(*a*, *t*|*S*) (again, when treated as a function of *t *with *a *and *S *held fixed). The quantity

|Va|=1d2ln⁡P(a,t|S)dt2|t=t^a     (5)
 MathType@MTEF@5@5@+=feaafiart1ev1aaatCvAUfKttLearuWrP9MDH5MBPbIqV92AaeXatLxBI9gBaebbnrfifHhDYfgasaacH8akY=wiFfYdH8Gipec8Eeeu0xXdbba9frFj0=OqFfea0dXdd9vqai=hGuQ8kuc9pgc9s8qqaq=dirpe0xb9q8qiLsFr0=vr0=vr0dc8meaabaqaciaacaGaaeqabaqabeGadaaakeaacqGG8baFcqWGwbGvdaWgaaWcbaGaemyyaegabeaakiabcYha8jabg2da9maalaaabaGaeGymaedabaWaaSaaaeaacqWGKbazdaahaaWcbeqaaiabikdaYaaakiGbcYgaSjabc6gaUjabdcfaqjabcIcaOiabdggaHjabcYcaSiabdsha0jabcYha8jabdofatjabcMcaPaqaaiabdsgaKjabdsha0naaCaaaleqabaGaeGOmaidaaaaakiabcYha8jabdsha0jabg2da9iqbdsha0zaajaWaaSbaaSqaaiabdggaHbqabaaaaOGaaCzcaiaaxMaadaqadaqaaiabiwda1aGaayjkaiaawMcaaaaa@5187@

is the modal dispersion, which is the reciprocal of the curvature at the mode t^a
 MathType@MTEF@5@5@+=feaafiart1ev1aaatCvAUfKttLearuWrP9MDH5MBPbIqV92AaeXatLxBI9gBaebbnrfifHhDYfgasaacH8akY=wiFfYdH8Gipec8Eeeu0xXdbba9frFj0=OqFfea0dXdd9vqai=hGuQ8kuc9pgc9s8qqaq=dirpe0xb9q8qiLsFr0=vr0=vr0dc8meaabaqaciaacaGaaeqabaqabeGadaaakeaacuWG0baDgaqcamaaBaaaleaacqWGHbqyaeqaaaaa@2FA4@. We make a further approximation,

*P*(*a*|*S*) ≈ *P*(*a*, t^a
 MathType@MTEF@5@5@+=feaafiart1ev1aaatCvAUfKttLearuWrP9MDH5MBPbIqV92AaeXatLxBI9gBaebbnrfifHhDYfgasaacH8akY=wiFfYdH8Gipec8Eeeu0xXdbba9frFj0=OqFfea0dXdd9vqai=hGuQ8kuc9pgc9s8qqaq=dirpe0xb9q8qiLsFr0=vr0=vr0dc8meaabaqaciaacaGaaeqabaqabeGadaaakeaacuWG0baDgaqcamaaBaaaleaacqWGHbqyaeqaaaaa@2FA4@|*S*)*C(*S*)     (6)

where *C(S) *is a constant that depends on *S *but not on *a *or *t*. This approximation can be made when we wish to maximise *P*(*a*|*S*) over a set of *a *for which |*V*_*a*_| is approximately constant. The goodness of this approximation is discussed below.

Given that our approximations hold, Equation (6) shows that a^
 MathType@MTEF@5@5@+=feaafiart1ev1aaatCvAUfKttLearuWrP9MDH5MBPbIqV92AaeXatLxBI9gBaebbnrfifHhDYfgasaacH8akY=wiFfYdH8Gipec8Eeeu0xXdbba9frFj0=OqFfea0dXdd9vqai=hGuQ8kuc9pgc9s8qqaq=dirpe0xb9q8qiLsFr0=vr0=vr0dc8meaabaqaciaacaGaaeqabaqabeGadaaakeaacuWGHbqygaqcaaaa@2E07@ maximises *P*(*a*|*S*) if and only if a^
 MathType@MTEF@5@5@+=feaafiart1ev1aaatCvAUfKttLearuWrP9MDH5MBPbIqV92AaeXatLxBI9gBaebbnrfifHhDYfgasaacH8akY=wiFfYdH8Gipec8Eeeu0xXdbba9frFj0=OqFfea0dXdd9vqai=hGuQ8kuc9pgc9s8qqaq=dirpe0xb9q8qiLsFr0=vr0=vr0dc8meaabaqaciaacaGaaeqabaqabeGadaaakeaacuWGHbqygaqcaaaa@2E07@ maximises *P*(*a*,t^a
 MathType@MTEF@5@5@+=feaafiart1ev1aaatCvAUfKttLearuWrP9MDH5MBPbIqV92AaeXatLxBI9gBaebbnrfifHhDYfgasaacH8akY=wiFfYdH8Gipec8Eeeu0xXdbba9frFj0=OqFfea0dXdd9vqai=hGuQ8kuc9pgc9s8qqaq=dirpe0xb9q8qiLsFr0=vr0=vr0dc8meaabaqaciaacaGaaeqabaqabeGadaaakeaacuWG0baDgaqcamaaBaaaleaacqWGHbqyaeqaaaaa@2FA4@|*S*). Since by definition (a^
 MathType@MTEF@5@5@+=feaafiart1ev1aaatCvAUfKttLearuWrP9MDH5MBPbIqV92AaeXatLxBI9gBaebbnrfifHhDYfgasaacH8akY=wiFfYdH8Gipec8Eeeu0xXdbba9frFj0=OqFfea0dXdd9vqai=hGuQ8kuc9pgc9s8qqaq=dirpe0xb9q8qiLsFr0=vr0=vr0dc8meaabaqaciaacaGaaeqabaqabeGadaaakeaacuWGHbqygaqcaaaa@2E07@, t^a
 MathType@MTEF@5@5@+=feaafiart1ev1aaatCvAUfKttLearuWrP9MDH5MBPbIqV92AaeXatLxBI9gBaebbnrfifHhDYfgasaacH8akY=wiFfYdH8Gipec8Eeeu0xXdbba9frFj0=OqFfea0dXdd9vqai=hGuQ8kuc9pgc9s8qqaq=dirpe0xb9q8qiLsFr0=vr0=vr0dc8meaabaqaciaacaGaaeqabaqabeGadaaakeaacuWG0baDgaqcamaaBaaaleaacqWGHbqyaeqaaaaa@2FA4@) maximises *P*(*a*, *t*|*S*), we see that a^
 MathType@MTEF@5@5@+=feaafiart1ev1aaatCvAUfKttLearuWrP9MDH5MBPbIqV92AaeXatLxBI9gBaebbnrfifHhDYfgasaacH8akY=wiFfYdH8Gipec8Eeeu0xXdbba9frFj0=OqFfea0dXdd9vqai=hGuQ8kuc9pgc9s8qqaq=dirpe0xb9q8qiLsFr0=vr0=vr0dc8meaabaqaciaacaGaaeqabaqabeGadaaakeaacuWGHbqygaqcaaaa@2E07@ can be found by unrestricted optimisation of *P*(*a*, *t*|*S*). Our algorithm to find a^
 MathType@MTEF@5@5@+=feaafiart1ev1aaatCvAUfKttLearuWrP9MDH5MBPbIqV92AaeXatLxBI9gBaebbnrfifHhDYfgasaacH8akY=wiFfYdH8Gipec8Eeeu0xXdbba9frFj0=OqFfea0dXdd9vqai=hGuQ8kuc9pgc9s8qqaq=dirpe0xb9q8qiLsFr0=vr0=vr0dc8meaabaqaciaacaGaaeqabaqabeGadaaakeaacuWGHbqygaqcaaaa@2E07@ exploits the fact that we are free to solve the unrestricted optimisation problem with any manner we choose, and specifically that we can "change the order of maximisation". The statistical argument we presented above says that we should find t^a
 MathType@MTEF@5@5@+=feaafiart1ev1aaatCvAUfKttLearuWrP9MDH5MBPbIqV92AaeXatLxBI9gBaebbnrfifHhDYfgasaacH8akY=wiFfYdH8Gipec8Eeeu0xXdbba9frFj0=OqFfea0dXdd9vqai=hGuQ8kuc9pgc9s8qqaq=dirpe0xb9q8qiLsFr0=vr0=vr0dc8meaabaqaciaacaGaaeqabaqabeGadaaakeaacuWG0baDgaqcamaaBaaaleaacqWGHbqyaeqaaaaa@2FA4@ for each *a*, and then maximise *P*(*a*, t^a
 MathType@MTEF@5@5@+=feaafiart1ev1aaatCvAUfKttLearuWrP9MDH5MBPbIqV92AaeXatLxBI9gBaebbnrfifHhDYfgasaacH8akY=wiFfYdH8Gipec8Eeeu0xXdbba9frFj0=OqFfea0dXdd9vqai=hGuQ8kuc9pgc9s8qqaq=dirpe0xb9q8qiLsFr0=vr0=vr0dc8meaabaqaciaacaGaaeqabaqabeGadaaakeaacuWG0baDgaqcamaaBaaaleaacqWGHbqyaeqaaaaa@2FA4@|*S*) over all *a*. An equivalent solution is to find a^
 MathType@MTEF@5@5@+=feaafiart1ev1aaatCvAUfKttLearuWrP9MDH5MBPbIqV92AaeXatLxBI9gBaebbnrfifHhDYfgasaacH8akY=wiFfYdH8Gipec8Eeeu0xXdbba9frFj0=OqFfea0dXdd9vqai=hGuQ8kuc9pgc9s8qqaq=dirpe0xb9q8qiLsFr0=vr0=vr0dc8meaabaqaciaacaGaaeqabaqabeGadaaakeaacuWGHbqygaqcaaaa@2E07@_*t*_, for each *t *(that is, the best alignment for a given *t*) and then maximise *P*(a^
 MathType@MTEF@5@5@+=feaafiart1ev1aaatCvAUfKttLearuWrP9MDH5MBPbIqV92AaeXatLxBI9gBaebbnrfifHhDYfgasaacH8akY=wiFfYdH8Gipec8Eeeu0xXdbba9frFj0=OqFfea0dXdd9vqai=hGuQ8kuc9pgc9s8qqaq=dirpe0xb9q8qiLsFr0=vr0=vr0dc8meaabaqaciaacaGaaeqabaqabeGadaaakeaacuWGHbqygaqcaaaa@2E07@_*t*_, *t*|*S*) over all *t*. The second solution is much easier in practice, because a^
 MathType@MTEF@5@5@+=feaafiart1ev1aaatCvAUfKttLearuWrP9MDH5MBPbIqV92AaeXatLxBI9gBaebbnrfifHhDYfgasaacH8akY=wiFfYdH8Gipec8Eeeu0xXdbba9frFj0=OqFfea0dXdd9vqai=hGuQ8kuc9pgc9s8qqaq=dirpe0xb9q8qiLsFr0=vr0=vr0dc8meaabaqaciaacaGaaeqabaqabeGadaaakeaacuWGHbqygaqcaaaa@2E07@_*t*_, can be found using a standard dynamic programming algorithm for pair HMMs [21], and then *P*(a^
 MathType@MTEF@5@5@+=feaafiart1ev1aaatCvAUfKttLearuWrP9MDH5MBPbIqV92AaeXatLxBI9gBaebbnrfifHhDYfgasaacH8akY=wiFfYdH8Gipec8Eeeu0xXdbba9frFj0=OqFfea0dXdd9vqai=hGuQ8kuc9pgc9s8qqaq=dirpe0xb9q8qiLsFr0=vr0=vr0dc8meaabaqaciaacaGaaeqabaqabeGadaaakeaacuWGHbqygaqcaaaa@2E07@_*t*_, *t*|*S*) can be maximised using any standard algorithm for maximising a one dimensional function.

The dynamic programming algorithm guarantees to find the global maximising a^
 MathType@MTEF@5@5@+=feaafiart1ev1aaatCvAUfKttLearuWrP9MDH5MBPbIqV92AaeXatLxBI9gBaebbnrfifHhDYfgasaacH8akY=wiFfYdH8Gipec8Eeeu0xXdbba9frFj0=OqFfea0dXdd9vqai=hGuQ8kuc9pgc9s8qqaq=dirpe0xb9q8qiLsFr0=vr0=vr0dc8meaabaqaciaacaGaaeqabaqabeGadaaakeaacuWGHbqygaqcaaaa@2E07@_*t *_(with ties broken arbitrarily). We find a straightforward Golden Section Search [28] to be adequate for maximising *P*(a^
 MathType@MTEF@5@5@+=feaafiart1ev1aaatCvAUfKttLearuWrP9MDH5MBPbIqV92AaeXatLxBI9gBaebbnrfifHhDYfgasaacH8akY=wiFfYdH8Gipec8Eeeu0xXdbba9frFj0=OqFfea0dXdd9vqai=hGuQ8kuc9pgc9s8qqaq=dirpe0xb9q8qiLsFr0=vr0=vr0dc8meaabaqaciaacaGaaeqabaqabeGadaaakeaacuWGHbqygaqcaaaa@2E07@_*t*_, *t*|*S*). This assumes that there is a single global optimum to be found. Actually we are able to trap events where local optima are detected. However, no local optima has ever been detected. We terminate the search when the values of *t *bracketing the maximum differ by less than 0.001. Moreover, we are able to terminate earlier when the optimal alignment is the same at all points within the bracketing area.

#### Parameterization of models of sequence evolution

Our model of noncoding DNA evolution is parameterized according to the empirical distribution of indel lengths and their overall rate relative to nucleotide substitutions from species for which essentially unambiguous alignments can be made. Here, we consider a parameterization by intronic data of *D. simulans *and *D. sechellia *(Shown in Figure 2). For these data, the rate of indels per interbase site, relative to the rate of nucleotide substitution, was previously estimated as θ = 0.225 [13]. We fitted the observed frequencies of different indels lengths to our model as follows. We directly use the observed frequencies of 1-bp and 2-bp indels, that is, 0.455 and 0.182, respectively. For indels of ≥ 3-bp, the frequencies, *W*_*x*_, for the model were obtained by minimizing the sum over ≥ 3-bp indels of the squared differences between the observed frequency distribution and *w*_*x *_= β/α^*x*^. Here β is a constant. The estimate for α was 1.170. Our software performs this curve fitting and in fact the whole analysis with a supplied empirical distribution of containing any lengths.

A GTR model of nucleotide substitution was fitted to *Drosophila *data shown in Table 1. By assuming the GTR model, we can then symmetrise this matrix by averaging the table with its transpose before any of the following calculations were carried out. The estimated equilibrium frequencies of each base are obtained from the normalised column sums, yielding (*q*_*A*_, *q*_*G*_, *q*_*C*_, *q*_*T*_) = (0.324, 0.197, 0.213, 0.266). The estimated rates of each type of substitution are obtained by dividing the entries in each column by the respective column sums, yielding:

Q^={0.9342010.04617120.02037620.0296080.02810140.9268020.01044930.01167640.01336530.01126130.9383490.02460380.02433170.01576580.03082550.934112}     (7)
 MathType@MTEF@5@5@+=feaafiart1ev1aaatCvAUfKttLearuWrP9MDH5MBPbIqV92AaeXatLxBI9gBaebbnrfifHhDYfgasaacH8akY=wiFfYdH8Gipec8Eeeu0xXdbba9frFj0=OqFfea0dXdd9vqai=hGuQ8kuc9pgc9s8qqaq=dirpe0xb9q8qiLsFr0=vr0=vr0dc8meaabaqaciaacaGaaeqabaqabeGadaaakeaacuWGrbqugaqcaiabg2da9maacmaabaqbaeqabqabaaaaaeaacqaIWaamcqGGUaGlcqaI5aqocqaIZaWmcqaI0aancqaIYaGmcqaIWaamcqaIXaqmaeaacqaIWaamcqGGUaGlcqaIWaamcqaI0aancqaI2aGncqaIXaqmcqaI3aWncqaIXaqmcqaIYaGmaeaacqaIWaamcqGGUaGlcqaIWaamcqaIYaGmcqaIWaamcqaIZaWmcqaI3aWncqaI2aGncqaIYaGmaeaacqaIWaamcqGGUaGlcqaIWaamcqaIYaGmcqaI5aqocqaI2aGncqaIWaamcqaI4aaoaeaacqaIWaamcqGGUaGlcqaIWaamcqaIYaGmcqaI4aaocqaIXaqmcqaIWaamcqaIXaqmcqaI0aanaeaacqaIWaamcqGGUaGlcqaI5aqocqaIYaGmcqaI2aGncqaI4aaocqaIWaamcqaIYaGmaeaacqaIWaamcqGGUaGlcqaIWaamcqaIXaqmcqaIWaamcqaI0aancqaI0aancqaI5aqocqaIZaWmaeaacqaIWaamcqGGUaGlcqaIWaamcqaIXaqmcqaIXaqmcqaI2aGncqaI3aWncqaI2aGncqaI0aanaeaacqaIWaamcqGGUaGlcqaIWaamcqaIXaqmcqaIZaWmcqaIZaWmcqaI2aGncqaI1aqncqaIZaWmaeaacqaIWaamcqGGUaGlcqaIWaamcqaIXaqmcqaIXaqmcqaIYaGmcqaI2aGncqaIXaqmcqaIZaWmaeaacqaIWaamcqGGUaGlcqaI5aqocqaIZaWmcqaI4aaocqaIZaWmcqaI0aancqaI5aqoaeaacqaIWaamcqGGUaGlcqaIWaamcqaIYaGmcqaI0aancqaI2aGncqaIWaamcqaIZaWmcqaI4aaoaeaacqaIWaamcqGGUaGlcqaIWaamcqaIYaGmcqaI0aancqaIZaWmcqaIZaWmcqaIXaqmcqaI3aWnaeaacqaIWaamcqGGUaGlcqaIWaamcqaIXaqmcqaI1aqncqaI3aWncqaI2aGncqaI1aqncqaI4aaoaeaacqaIWaamcqGGUaGlcqaIWaamcqaIZaWmcqaIWaamcqaI4aaocqaIYaGmcqaI1aqncqaI1aqnaeaacqaIWaamcqGGUaGlcqaI5aqocqaIZaWmcqaI0aancqaIXaqmcqaIXaqmcqaIYaGmaaaacaGL7bGaayzFaaGaaCzcaiaaxMaadaqadaqaaiabiEda3aGaayjkaiaawMcaaaaa@B877@

Finally, find the matrix *A *that satisfies Equation (3) when time is measured in units of expected substitutions, to obtain our estimate of the instantaneous rate matrix:

A^={−0.9951070.7069880.3012770.4468170.430299−1.10370.1534140.171360.1976160.165335−0.9221520.3731110.3671920.2313750.467461−0.991288}.     (8)
 MathType@MTEF@5@5@+=feaafiart1ev1aaatCvAUfKttLearuWrP9MDH5MBPbIqV92AaeXatLxBI9gBaebbnrfifHhDYfgasaacH8akY=wiFfYdH8Gipec8Eeeu0xXdbba9frFj0=OqFfea0dXdd9vqai=hGuQ8kuc9pgc9s8qqaq=dirpe0xb9q8qiLsFr0=vr0=vr0dc8meaabaqaciaacaGaaeqabaqabeGadaaakeaacuWGbbqqgaqcaiabg2da9maacmaabaqbaeqabqabaaaaaeaacqGHsislcqaIWaamcqGGUaGlcqaI5aqocqaI5aqocqaI1aqncqaIXaqmcqaIWaamcqaI3aWnaeaacqaIWaamcqGGUaGlcqaI3aWncqaIWaamcqaI2aGncqaI5aqocqaI4aaocqaI4aaoaeaacqaIWaamcqGGUaGlcqaIZaWmcqaIWaamcqaIXaqmcqaIYaGmcqaI3aWncqaI3aWnaeaacqaIWaamcqGGUaGlcqaI0aancqaI0aancqaI2aGncqaI4aaocqaIXaqmcqaI3aWnaeaacqaIWaamcqGGUaGlcqaI0aancqaIZaWmcqaIWaamcqaIYaGmcqaI5aqocqaI5aqoaeaacqGHsislcqaIXaqmcqGGUaGlcqaIXaqmcqaIWaamcqaIZaWmcqaI3aWnaeaacqaIWaamcqGGUaGlcqaIXaqmcqaI1aqncqaIZaWmcqaI0aancqaIXaqmcqaI0aanaeaacqaIWaamcqGGUaGlcqaIXaqmcqaI3aWncqaIXaqmcqaIZaWmcqaI2aGnaeaacqaIWaamcqGGUaGlcqaIXaqmcqaI5aqocqaI3aWncqaI2aGncqaIXaqmcqaI2aGnaeaacqaIWaamcqGGUaGlcqaIXaqmcqaI2aGncqaI1aqncqaIZaWmcqaIZaWmcqaI1aqnaeaacqGHsislcqaIWaamcqGGUaGlcqaI5aqocqaIYaGmcqaIYaGmcqaIXaqmcqaI1aqncqaIYaGmaeaacqaIWaamcqGGUaGlcqaIZaWmcqaI3aWncqaIZaWmcqaIXaqmcqaIXaqmcqaIXaqmaeaacqaIWaamcqGGUaGlcqaIZaWmcqaI2aGncqaI3aWncqaIXaqmcqaI5aqocqaIYaGmaeaacqaIWaamcqGGUaGlcqaIYaGmcqaIZaWmcqaIXaqmcqaIZaWmcqaI3aWncqaI1aqnaeaacqaIWaamcqGGUaGlcqaI0aancqaI2aGncqaI3aWncqaI0aancqaI2aGncqaIXaqmaeaacqGHsislcqaIWaamcqGGUaGlcqaI5aqocqaI5aqocqaIXaqmcqaIYaGmcqaI4aaocqaI4aaoaaaacaGL7bGaayzFaaGaeiOla4IaaCzcaiaaxMaadaqadaqaaiabiIda4aGaayjkaiaawMcaaaaa@B033@

#### Performance evaluation

For non-coding sequences, there are few externally verified alignments available to test the performance of alignment methods. As a substitute, we simulate sequence divergence *in silico*, so that sequences are generated that are related by a known, "correct" alignment [7]. We tested the MCALIGN2 program by examining the posterior probability of the best alignment found by the algorithm, the fraction of correctly aligned sites, an estimate of divergence time calculated from the estimated alignment, and the time taken to compute the alignment.

We compared the dynamic programming approach used here against the Monte-Carlo approach proposed previously [13] and the pair HMM approach of Knudsen and Miyamoto [11] in simulations assuming the Jukes-Cantor model of nucleotide evolution. In comparisons of MCALING2 and MCALIGN, for each simulated pair of sequences, we compared the posterior probability, *P*(*a*|*S*) ≃ *P*(*a*, t^a
 MathType@MTEF@5@5@+=feaafiart1ev1aaatCvAUfKttLearuWrP9MDH5MBPbIqV92AaeXatLxBI9gBaebbnrfifHhDYfgasaacH8akY=wiFfYdH8Gipec8Eeeu0xXdbba9frFj0=OqFfea0dXdd9vqai=hGuQ8kuc9pgc9s8qqaq=dirpe0xb9q8qiLsFr0=vr0=vr0dc8meaabaqaciaacaGaaeqabaqabeGadaaakeaacuWG0baDgaqcamaaBaaaleaacqWGHbqyaeqaaaaa@2FA4@|*S*), of the best alignment found by MCALIGN2 with the best alignments found by MCALIGN.

We also compared MCALIGN2 against AVID of Bray et al. [17] in simulations assuming a GTR model, parameterised using the *Drosophila *intronic data as described above. In these comparisons we investigated cases in which the model assumed by MCALIGN2 differed from the simulation model, by using the simpler JC and K2P models to analyse data simulated under a GTR model.

In all comparisons, we calculated the fraction of correctly aligned sites by counting the number of base pairs or bases-to-gaps which were correctly aligned in a comparison to the true alignment. As an alternative measure of alignment quality, we considered the precision of divergence time estimated from the alignments. The estimator of divergence time we used was distance under the GTR model. It is made by estimating the base frequencies *q*_*i*_, and the rates *a*_*ij*_, and finding ones that most closely predict the observed net transition matrix *P *[26]. This estimator of divergence time uses only the non-indel regions, and does not use the presence of indels to help estimate divergence time. For all the simulations with a given divergence time *t *and a certain evolutionary model, we observed the mean and variance of the estimator of *t *calculated from both the true alignment and the alignments estimated by sequence alignment methods we considered here. We express the precision of the estimator as the estimated root mean squared error (e.r.m.s.e.), since none of the estimators examined are perfectly unbiased. For *t*, this is

e.r.m.s.e.=1N∑(test−ttrue)2     (9)
 MathType@MTEF@5@5@+=feaafiart1ev1aaatCvAUfKttLearuWrP9MDH5MBPbIqV92AaeXatLxBI9gBaebbnrfifHhDYfgasaacH8akY=wiFfYdH8Gipec8Eeeu0xXdbba9frFj0=OqFfea0dXdd9vqai=hGuQ8kuc9pgc9s8qqaq=dirpe0xb9q8qiLsFr0=vr0=vr0dc8meaabaqaciaacaGaaeqabaqabeGadaaakeaacqWGLbqzcqGGUaGlcqWGYbGCcqGGUaGlcqWGTbqBcqGGUaGlcqWGZbWCcqGGUaGlcqWGLbqzcqGGUaGlcqGH9aqpdaGcaaqaamaalaaabaGaeGymaedabaGaemOta4eaamaaqaeabaWaaeWaaeaacqWG0baDdaWgaaWcbaGaemyzauMaem4CamNaemiDaqhabeaakiabgkHiTiabdsha0naaBaaaleaacqWG0baDcqWGYbGCcqWG1bqDcqWGLbqzaeqaaaGccaGLOaGaayzkaaWaaWbaaSqabeaacqaIYaGmaaaabeqab0GaeyyeIuoaaSqabaGccaWLjaGaaCzcamaabmaabaGaeGyoaKdacaGLOaGaayzkaaaaaa@51E5@

when there are *N *simulations.

Although our program allows any prior for divergence time, for all comparisons we used a relatively diffuse or uninformative prior:

P(t)=43e(−43t)     (10)
 MathType@MTEF@5@5@+=feaafiart1ev1aaatCvAUfKttLearuWrP9MDH5MBPbIqV92AaeXatLxBI9gBaebbnrfifHhDYfgasaacH8akY=wiFfYdH8Gipec8Eeeu0xXdbba9frFj0=OqFfea0dXdd9vqai=hGuQ8kuc9pgc9s8qqaq=dirpe0xb9q8qiLsFr0=vr0=vr0dc8meaabaqaciaacaGaaeqabaqabeGadaaakeaacqWGqbaucqGGOaakcqWG0baDcqGGPaqkcqGH9aqpdaWcaaqaaiabisda0aqaaiabiodaZaaacqqGLbqzdaahaaWcbeqaaiabcIcaOiabgkHiTmaalaaabaGaeGinaqdabaGaeG4mamdaaiabdsha0jabcMcaPaaakiaaxMaacaWLjaWaaeWaaeaacqaIXaqmcqaIWaamaiaawIcacaGLPaaaaaa@4035@

which has the mean 0.75. Because low divergences are more likely than high ones for two homologous sequences, this prior on *t *seems to be a reasonable one.

## Results

### Comparison amongst PairHMM_KM, MCALIGN2(DP) and MCALIGN(MC)

We generated non-coding sequence data using a model of non-coding DNA evolution in which gap lengths are parametrized by intronic data of *D. simulans *and *D. sechellia*, and point substitutions occur according to the Jukes-Cantor model to compare the performances of PairHMM_KM, MCALIGN2 (DP hereafter) and MCALIGN (MC hereafter). In this setting, the DP and MC methods aim to find the same most probable alignment, since they assume essentially the same model and prior, but use different algorithms.

Table 2 and 3 show the mean and e.r.m.s.e. of estimated divergence time (*t*), and the proportions of correctly aligned sites for combinations of θ and *t*. All alignment methods perform similarly when the true divergence time is not too great, *t *≤ 0.2, and the indel rate is not too great, θ ≤ 0.3. For these parameters, the fraction of correctly aligned bases is greater than 90% and is similar for all the three methods. The mean estimated divergence time calculated from estimated alignments are close to the true values, and the e.r.m.s.e. are not substantially greater than if the true alignment is known. However, when the divergence time *t *became larger (*t *> 0.2) or the indel rate becomes larger (θ = 0.4), the performance of the MC method becomes noticeably inferior, since the mean proportion of correctly aligned bases is significantly lower than for the alignments estimated by the DP and PairHMM_KM method, and the divergence time estimates are more biased and have larger e.r.m.s.e. For the largest indel ratio we considered, θ = 0.4, the MC method tends to estimate an alignment with too many gaps and the estimates of *t *tend to be higher than the true values. Table 3 also shows that the DP and PairHMM_KM methods both have more stable performances for most of the cases we have considered, in the sense of producing lower standard deviations of proportions of correctly aligned sites. It is also shown that the efficiency of MCALIGN2 is generally slightly better than PairHMM_KM.

**Figure 3:**
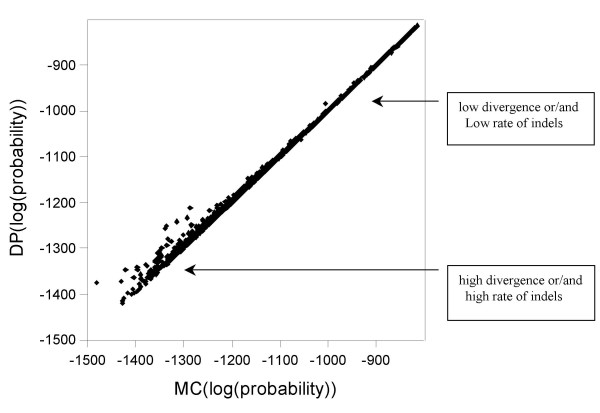
Probability test using the probability function of MCALIGN2(DP), comparing the performances of alignments produced by the DP method and the MC method. All the values here are log values.

For the same simulated datasets, Figure 3 compares the the log values of alignment probability for MCALIGN2 and MCALIGN, since they use essentially the same scoring function. For the two methods, the approximation of Equation (6) was used to calculate alignment probability marginal to divergence time. Both methods perform equivalently for almost all the simulations when divergence time is very small (*t *= 0.05); we presume that both methods are able to find the globally most probable alignment. However, when divergence time and/or rate of indel events becomes larger, the DP method begins to outperform the MC method, in the sense that the alignments produced by MCALIGN2 have higher probabilities. For the highest divergence time (*t *= 0.30) and/or rate of indel events (θ = 0.40) we considered, the DP method outperformed the MC method for almost all of the replicate simulations. This clearly indicates that the MC algorithm of Keightley and Johnson [13] gets stuck at local optima.

### Comparison between MCALIGN2 and AVID

For each combination of values of *t *and θ, 200 replicate simulations were performed, each simulating a pair of sequences of length 500 base pairs, evolving under an indel model and a general time reversible (GTR) model of nucleotide substitution, parameterised using real Drosophila data. This model is very different to the simple Jukes-Cantor (JC) model, and quite different to Kimura's 2 parameter (K2P) model. In addition to comparing MCALIGN2 with AVID, it is interesting to explore the effect of the nucleotide substitution model assumed by MCALIGN2. We aligned each simulated pair of sequences using MCALIGN2 under the assumptions of the correct GTR model, a simple K2P model with the ratio of transition events to transversion events equal to 2, and the JC model.

The results in Table 4 and 5 show that the alignments found by MCALIGN2, when the correct GTR model was assumed, are more accurate for almost all combinations of parameter values we have considered. In comparison, alignments found by MCALIGN2, when the incorrect JC or K2P models were assumed, are only slightly less accurate. Alignments found by AVID generally have the lowest accuracy in the cases studied.

Here, lower accuracy is indicated by a lower proportion of correctly aligned bases, and estimates of divergence time *t *that are more biased and have larger e.r.m.s.e.. In particular, alignments produced by AVID exhibit consistent upward bias estimates of *t*, and lower means proportions of correctly aligned bases than alignments produced by MCAL1GN2. This remains true, for most of the cases we considered here, whether MCALIGN2 used the correct GTR model of nucleotide substitution, or the incorrect JC or K2P models. The improvement in alignment quality gained by knowing the correct model of nucleotide substitution is generally modest, but worthwhile.

### Test using real data

We also compared MCALIGN2 with AVID and CLUSTALW using real intronic DNA sequences from mouse and rat. Although we do not know the true alignments for real sequence data, we can still judge the alignment performances of different methods by examining the plausibility of the alignments (e.g. positions of gaps in the alignments and proportion of matched bases). Here we show three specific cases in which MCALIGN2 performed quite differently from AVID and CLUSTALW.

**Figure 4:**
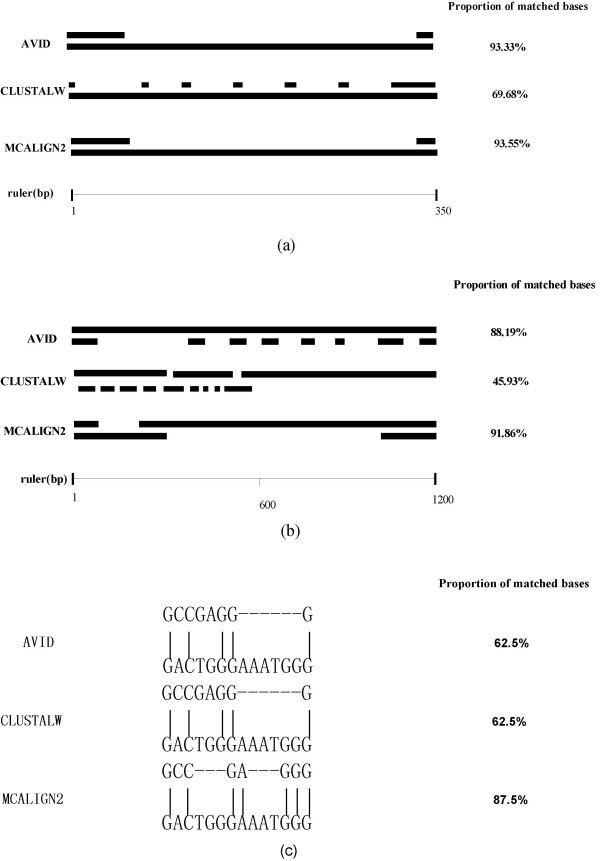
Alignments of real non-coding DNA sequences from mouse and rat produced by AVID, CLUSTALW and MCALIGN2. The scale is shown below the alignments, and proportion of matched bases in the alignment is shown on the right. There are three cases (a) alignments of intronic DNA sequence from the *Fshb *gene, (b) alignments of intronic DNA sequences from the *Omd *gene and (c) alignments of small pieces of intronic DNA sequences from *Omd*, in which MCALIGN2 performed quite differently from the others.

As shown in Figure 4(a), AVID and MCALIGN2 produced similar alignments, which include a long gap between ~70 bp–~320 bp. However, the alignment produced by CLUSTALW has several small gaps, which are separated by small segments of aligned bases. In this example, 93% of base pairs are matched in alignments produced by AVID and MCALIGN2, while only 70% of base pairs are matched in the alignment produced by CLUSTALW. Although it is impossible to say which alignment is the true alignment, the positions of gaps and proportion of matched bases can give some indications of the alignment plausibility. As the gap-open penalty is higher than the gap-extension penalty, the cost of having several small gaps is higher than the cost of having a long gap, if the total length of gaps is similar among different alignments. Meanwhile, as the match state has a positive effect on the alignment probability, the alignment with the higher proportion of matched bases is more likely to be correct. Therefore, from the point view of the alignment probability, the alignments produced by MCALIGN2 and AVID in this case are more plausible.

Figure 4(b) shows a different fragment from alignments produced by AVID, CLUSTALW and MCALIGN2. In this case, the alignment produced by MCALIGN2 also has a long gap from ~300 bp–~1000 bp, and it has the highest proportion of matched bases compared to other alignments. However, the alignment produced by CLUSTALW has several small gaps and a long gap in the terminal portion, and it has the lowest proportion of matched bases. Although the alignment produced by AVID looks better than the one produced by CLUSTALW, it is still fragmented by several small-length gaps.

However, MCALIGN2 does not always produce less gaps than other methods. As shown in Figure 4(c), the alignment produced by MCALIGN2 has more gaps than the others, but a smaller number of nucleotide differences. Without other information it is impossible to say which is more plausible.

### Execution time

**Figure 5:**
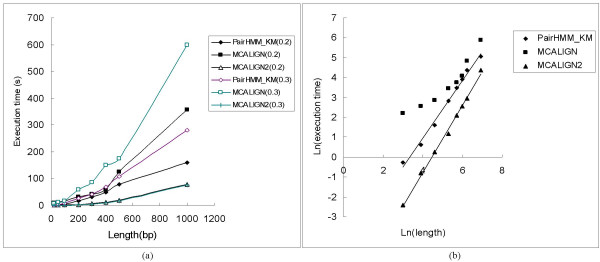
Execution time plotted against sequence length for sequence divergence of 0.2 and 0.3, and ratio of indels of 0.225. Execution times were estimated from the average of ten simulations. (a) execution time of MCALIGN2 comparing to MCALIGN and the pair HMM method of Knudsen and Miyamoto (black points and lines for divergence of 0.2; colored points and lines for divergence of 0.3). (b) tests the correctness of the program based on execution time for sequence divergence of 0.2. All the numbers are in natural log. The slope of log(time) against log(length) is 1.91 for MCALIGN2, which means the program closely follows the expected algorithm time operation O(n^2^). The slope of log(time) against log(length) for PairHMM_KM is 1.78, not far away from the order n^2^. The pattern for MCALIGN is hard to track.

Figure 5 shows the execution times of the different alignment algorithms tested above, as a function of sequence length. Execution times were measured in on a 2.8 GHz Intel^® ^Xeon™ processor. Results are shown for divergence time *t *= 0.2 and 0.3, and ratio of indels to point substitutions θ = 0.225. Figure 5 shows that for the DP method (MCALIGN2), execution time increases as a quadratic function of sequence length, as expected. Similar behaviour is observed for the PairHMM_KM method, since this calculates the sum of the probabilities of all alignments for two given sequences using the forward algorithm for pair HMMs, and this gives a time and memory complexity on the order of *L*^*2 *^[11]. For the MC method (MCALIGN) execution time increases with sequence length, and, although it does not follow a power law, it is roughly quadratic for long sequences. Although it shows a substantial improvement over the previous Monte Carlo method and it is faster than the PairHMM_KM method, MCALIGN2 still cannot compare with a heuristic alignment method such as AVID. To align two 1000-bp sequences (*t *= 0.2) takes about 0.1s using AVID, about 80s using MCALIGN2, about 160s using PairHMM_KM and about 350s using MCALIGN. Furthermore, it is also shown in Figure 5(a) that both MCALIGN and PairHMM_KM take longer to align sequences with larger divergence times, whereas execution time of MCALIGN2 is unaffected by divergence. However, given the pair HMM model used in PairHMM_KM, execution time should not be affected by divergence for this method. We suppose that this occurred due to small tolerances chosen for ML estimation of divergence time in this program.

## Discussion

The problem of statistical inference of an alignment can be separated into two parts: specifying a scoring function, and finding an alignment that optimises that scoring function. The scoring function is specified on biological and/or statistical grounds, and determines the biological meaningfulness and accuracy of the inferred alignment. The choice of optimising algorithm determines the speed of the method, and may hamper accuracy if convergence to a global optimum cannot be guaranteed. A useful alignment method must produce biologically meaningful and accurate alignments, and also must do so quickly. There is a trade-off because the most biologically realistic scoring functions are difficult to optimise.

Many scoring functions can essentially be described by the relative contributions for individual nucleotide substitution and indel events, which were traditionally thought of as penalty scores for mismatches and for gaps. However, no general theory guides the selection of these penalties [31], unless divergence time is known [21]. Although almost all scoring functions have a *probabilistic *interpretation [21], only ones in which divergence time is an explicit parameter have an *evolutionary *interpretation. This inclusion of a time parameter is crucial in allowing us to train or parameterize our model using closely related sequences, in order to improve the accuracy of alignments between more distantly related sequences. Although the idea of training a scoring function on known alignments is an old one (especially with respect to amino acid substitutions (e.g. PAM250 matrix of Dayhoff et al. [32])), in the past it has generally been necessary to use a training set of sequences at similar evolutionary distance as the sequences that are ultimately to be aligned.

Heuristic scoring functions are often chosen because an algorithm exists to optimise them efficiently. However, without any underlying evolutionary model, the alignments produced by such methods will be biased (at least at some evolutionary distances), in the sense that they will exhibit features that depart in a systematic direction from the true alignment.

The evolutionary model used in our method strikes a balance between biological realism and computational tractability. We ignore multiple hits of indel events, and assume a distribution of indel lengths that corresponds to an improved affine gap penalty scheme. Our model is therefore quite different from more realistic evolutionary models that account properly for multiple hits of indel events [8,9,11,12]. The TKF91 model is particularly unrealistic for non-coding DNA, since it allows only single base indels. Keightley and Johnson [13] suggest that the present model (ignoring multiple hits for indels) is a better approximation to their simulation model (which allowed multiple hits of multi-base pair indels), for the parameter values used in their simulations. The TKF92 model allows a geometric distribution of indel lengths, but only allows whole insertions to be subsequently deleted, or vice versa. That model has therefore been criticised as introducing non-biological "hidden fragment boundaries". Since our model does not allow insertions to be deleted at all, or vice versa, it could be seen as also introducing "hidden fragment boundaries". Our model allows a more realistic distribution of indel lengths than the TKF92 model. The approach of Knudsen and Miyamoto [11] could be seen as an extension of the TKF92 model, assuming a geometric distribution of indel lengths and allowing multiple hits involving up to two indel events. Our results suggest that this model (approximated using a three state pair HMM), and our model (using a seven state HMM) offer approximations of very similar quality. Intuitively, we would have expected our model to be superior when multiple hits of indel events were rare, i.e. for relatively smaller evolutionary distances and indel rates. However, it seems that in such cases the performance of both methods is so good that it is hard to detect any difference. The "long indel" model of Miklos et al. [12] is certainly more realistic than either model, since it allows an arbitrary distribution of indel lengths and accounts almost exactly for multiple hits of indels. However, the finite trajectory algorithm [12] used to account for multiple hits is computationally expensive (O(L^4^) in complexity).

When comparing the present method (MCALIGN2) against a previous Monte Carlo approach (MCALIGN [13]) we are comparing the performance of two different optimisers, with the same scoring function. Generally MCALIGN2 has better global optimum finding properties, and is much faster than the Monte Carlo method to align the same sequences. There are two major reasons for this improvement:

(i) MCALIGN2 uses a dynamic programming algorithm that is guaranteed to find the most probable alignment for a given divergence time, whereas the stochastic hill-climbing algorithm used in the Monte Carlo method can only search locally by making heuristically chosen adjustments to an alignment.

(ii) MCALIGN2 stops its search when the maximising divergence time is bracketed to high precision, with the bracket length being reduced by a geometric factor at each step of the algorithm. In contrast, the Monte Carlo method must search until no improvement in alignment probability is found during a predetermined number of iterations.

In comparisons of MCALIGN2 against the pair HMM method of Knudsen and Miyamoto, a method with an evolutionary time parameter and an affine gap penalty [11], we found that the two methods performed very similarly for almost all cases, but MCALIGN2 is computationally faster. When comparing MCALIGN2 against AVID, a time-naive model [17], we found that MCALIGN2 produced better quality alignments than AVID for almost all combinations of parameters. This shows that, when the evolutionary model is known, this knowledge can be used in in a model based inference method to estimate alignment more accurately.

Despite being substantially faster than our original Monte Carlo approach and the pair HMM method of Knudsen and Miyamoto, MCALIGN2 cannot compete with AVID in terms of execution time, because of the clever heuristics used by AVID. Its general strategy for aligning two sequences is to select anchors using a variant of the Smith-Waterman algorithm [33] to split long sequences into short sequences, which are aligned by a dynamic programming algorithm, Needleman-Wunsch [34]. A set of maximal matches between sequences is constructed using a suffix tree. This approach is fast and memory efficient, and practical for sequence alignments of large genomic regions up to megabases long [17]. In principle, the fast heuristics used by AVID can be applied for any pair HMM, and therefore could be combined with our approach to give faster, high quality alignments.

In order to examine the robustness of the MCALIGN2 method, we also investigated cases in which the model assumed in the MCALIGN2 analysis was a simpler model (JC or K2P) than the model the data were simulated under (GTR). Generally, the MCALIGN2 method assuming an incorrect model still has good performance for small and medium divergence times, but for larger divergence times and/or higher indel rates, performance suffers slightly compared with when the correct GTR model was assumed. Therefore, when aligning sequences from distant species, it is desirable to use an evolutionary model that is as realistic as possible. However, it is in precisely this situation that it may be most difficult to estimate a model, because the assumption of that the evolutionary process is the same between closely and distantly related species is most likely to break down.

When inferring alignment in a Bayesian framework, divergence time is a nuisance parameter that must be eliminated by integration (Equation 2). The computational implementation of our method relies totally on being able to approximate this integral (Equations 4 and 6) rather than having to calculate it numerically using e.g. quadrature. The approximations we make will be good when *P*(*t*|*a,S*) is approximately normal with constant variance for a certain set of high probability alignments. Because *P*(*t*|*a,S*) is a product of multinomial probabilities, the normality approximation will be good for long sequences under most models of molecular evolution. The assumption of constant variance will be reasonable when high probability alignments differ from each other by only a few indels and substitutions, relative to the total sequence length. As a concrete check of this assumption, we used the Monte Carlo search algorithm of Keightley and Johnson [13] and retained the set of all alignments visited that had probability at least 0.01 as large as the maximum probability. Within this set, the correlation between *P*(*a*|*S*) computed "exactly" (using quadrature) and *P*(*a*|*S*) exceeded 0.98.

It is worth mentioning that, to our knowledge, no better method has been found for eliminating divergence time as a nuisance parameter when estimating alignment. Most authors concentrate on finding the true MLE for *t*, summing over all possible alignments, using the EM algorithm [8-10,35]. The best way to estimate the alignment has not been considered in detail, but a common approach is to use the most probable alignment conditional on the observed sequences and conditional on the MLE for *t*. Although our method has a more direct Bayesian justification, given the approximations made it is likely that the two approaches will give similar results.

## Conclusion

Sequence alignment is a major issue for the evolutionary analysis of non-coding DNA. We developed a model-based method, MCALIGN2, as an improvement to the previous Monte Carlo method MCALIGN. MCALIGN2 uses a deterministic global optimiser to find the alignment with the highest posterior probability. It allows a rich class of evolutionary models of indel length along with any time reversible model of nucleotide substitution. As shown in the test results, MCALIGN2 outperforms other available non-coding DNA sequence alignment methods for all the cases we have considered.

## Availability and requirements

Project name: MCALIGN2

Project home page: 

Operating system: Platform independent

Programming language: C++

Other requirements: C++ compiler if downloading and compiling the source code

Licence: FSF GENERAL public licence.

## Authors' contributions

JW developed and tested the software in this study, and wrote the manuscript. PDK participated in the design of the study and wrote the manuscript. TJ initially conceived of and co-designed the software and wrote the manuscript. All authors read and approved the final manuscript.

## Figures and Tables

**Table 1:** *Drosophila *intronic data that is used to derive a GTR model of DNA evolution.

		**Sequence 2**				
		A	G	C	T	total
**Sequence 1**	A	1363	45	18	54	1480
	G	37	823	9	17	886
	C	21	11	898	32	962
	T	17	11	27	1120	1175
	total	1438	890	952	1223	4503

Pairs of nucleotide for 4503 sites of sequence that has diverged according to a general-time-reversible (GTR) model, from real *Drosophila *intronic data. The columns are the bases in the first sequence. Here, we chose a long intron from *D. simulans *and *D. melanogaster*, aligned them using AVID, then counted the aligned sites regardless of gaps.

**Table 2:** Performance of MCALIGN2(DP), MCALIGN(MC) and PairHMM_KM compared by the estimator of divergenct time corrected by the Jukes-Cantor model

Simulated			Alignment Estimated		
t	Θ	Alignment Known	PairHMM_KM	MC	DP
0.05	0.225	0.0502 (0.0107)	0.0496 (0.0103)	0.0499 (0.0104)	0.0501 (0.0103)
0.10	0.225	0.0998 (0.0146)	0.0987 (0.0152)	0.0994 (0.0153)	0.0989 (0.0154)
0.15	0.225	0.1493 (0.0208)	0.1482 (0.0226)	0.1507 (0.0230)	0.1487 (0.0208)
0.20	0.225	0.2025 (0.0241)	0.1994 (0.0267)	0.2053 (0.0263)	0.2001 (0.0256)
0.25	0.225	0.2515 (0.0286)	0.2440 (0.0348)	0.2593 (0.0346)	0.2476 (0.0319)
0.30	0.225	0.3003 (0.0311)	0.2955 (0.0419)	0.3162 (0.0525)	0.2981 (0.0349)
0.15	0.10	0.1519 (0.0198)	0.1503 (0.0189)	0.1502 (0.0189)	0.1500 (0.0188)
0.15	0.30	0.1515 (0.0202)	0.1507 (0.0226)	0.1566 (0.0234)	0.1523 (0.0218)
0.15	0.40	0.1512 (0.0194)	0.1480 (0.0220)	0.1645 (0.0263)	0.1516 (0.0213)

Estimates of sequence divergence, *t*, and proportion of matched bases from 200 replicates for each combination of t and θ, with sequences of length 500 base pairs. Estimated root mean square error (e.r.m.s.e.) is shown after divergence time in parentheses.

**Table 3:** Performance of MCALIGN2(DP), MCALIGN(MC) and PairHMM_KM compared by examining the proportions of correctly aligned sites.

Simulated		Proportions of correctly aligned sites.		
t	Θ	PAIRHMM	MC	DP
0.05	0.225	0.992 (0.0072)	0.992 (0.0078)	0.993 (0.0074)
0.10	0.225	0.977 (0.0133)	0.977 (0.0137)	0.977 (0.0127)
0.15	0.225	0.954 (0.0210)	0.951 (0.0235)	0.955 (0.0186)
0.20	0.225	0.920 (0.0300)	0.915 (0.0345)	0.922 (0.0293)
0.25	0.225	0.868 (0.0416)	0.850 (0.0596)	0.869 (0.0433)
0.30	0.225	0.810 (0.0500)	0.761 (0.0853)	0.813 (0.0511)
0.15	0.10	0.984 (0.0116)	0.983 (0.0102)	0.983 (0.0108)
0.15	0.30	0.933 (0.0224)	0.925 (0.0292)	0.933 (0.0246)
0.15	0.40	0.905 (0.0325)	0.894 (0.0376)	0.906 (0.0329)

Proportion of matched bases from 200 replicates for each combination of t and θ, with sequences of length 500 base pairs. Standard deviation of mean is shown after the proportion of matched bases in parentheses.

**Table 4:** Performance of MCALIGN2 and AVID compared by proportions of correctly aligned sites based on a GTR model

Simulated		Proportion of matched bases			
t	Θ	AVID	MCALIGN2(JC)	MCALIGN2(K2P)	MCALIGN2(GTR)
0.05	0.225	0.991 (0.0085)	0.993 (0.0057)	0.993 (0.0057)	0.993 (0.0057)
0.10	0.225	0.973 (0.0141)	0.978 (0.0127)	0.979 (0.0127)	0.979 (0.0127)
0.15	0.225	0.946 (0.0283)	0.954 (0.0212)	0.956 (0.0184)	0.958 (0.0184)
0.20	0.225	0.904 (0.0325)	0.916 (0.0283)	0.920 (0.0269)	0.922 (0.0269)
0.25	0.225	0.852 (0.0452)	0.867 (0.0438)	0.873 (0.0410)	0.876 (0.0396)
0.30	0.225	0.795 (0.0566)	0.811 (0.0495)	0.824 (0.0481)	0.831 (0.0481)
0.15	0.10	0.980 (0.0141)	0.982 (0.0113)	0.982 (0.0113)	0.983 (0.0113)
0.15	0.30	0.913 (0.0283)	0.935 (0.0226)	0.936 (0.0212)	0.941 (0.0212)
0.15	0.40	0.876 (0.0354)	0.900 (0.0311)	0.905 (0.0297)	0.916 (0.0283)

Proportion of matched bases from 200 replicates for each combination of t and θ, with sequences of length 500 base pairs. Standard deviation of mean is shown after the proportion of matched bases in parentheses. Here MCALIGN2 is tested by assuming either the correct model of DNA evolution(GTR) or the incorrect models (JC and K2P).

**Table 5:** Performance of MCALIGN2 and AVID compared by estimator of divergence time based on a General Time-Reversible Model

Simulated			Alignment Estimated			
t	Θ	Alignment Known	AVID	MCALIGN2(JC)	MCALIGN2(K2P)	MCALIGN2(GTR)
0.05	0.225	0.0504 (0.0100)	0.0523 (0.0181)	0.0501 (0.0100)	0.0500 (0.0100)	0.0500 (0.0100)
0.10	0.225	0.0991 (0.0145)	0.1023 (0.0159)	0.0981 (0.0151)	0.0976 (0.0149)	0.0983 (0.0149)
0.15	0.225	0.1522 (0.0191)	0.1575 (0.0226)	0.1496 (0.0192)	0.1494 (0.0192)	0.1502 (0.0194)
0.20	0.225	0.2034 (0.0225)	0.2131 (0.0283)	0.1985 (0.0238)	0.1978 (0.0239)	0.2003 (0.0235)
0.25	0.225	0.2531 (0.0286)	0.2699 (0.0383)	0.2493 (0.0316)	0.2453 (0.0308)	0.2491 (0.0311)
0.30	0.225	0.3003 (0.0302)	0.3222 (0.0420)	0.2944 (0.0324)	0.2914 (0.0311)	0.2987 (0.0323)
0.15	0.10	0.1520 (0.0207)	0.1528 (0.0219)	0.1510 (0.0203)	0.1508 (0.0203)	0.1510 (0.0204)
0.15	0.30	0.1509 (0.0200)	0.1634 (0.0301)	0.1489 (0.0207)	0.1470 (0.0208)	0.1499 (0.0207)
0.15	0.40	0.1526 (0.0208)	0.1794 (0.0398)	0.1507 (0.0213)	0.1477 (0.0203)	0.1508 (0.0211)

Estimates of sequence divergence,*t*, from 200 replicates for each combination of *t *and θ, with sequences of Length 500 base pairs. Estimated root mean square error (e.r.m.s.e.) is shown after divergence time in parentheses. Here MCALIGN2 is tested by assuming either the correct model of DNA evolution(GTR) or the incorrect models (JC and K2P).
